# Oral microbiome dysbiosis among cigarette smokers and smokeless tobacco users compared to non-users

**DOI:** 10.1038/s41598-024-60730-2

**Published:** 2024-05-06

**Authors:** Suhana Chattopadhyay, Leena Malayil, Jessica Chopyk, Eoghan Smyth, Prachi Kulkarni, Greg Raspanti, Stephen B. Thomas, Amir Sapkota, Emmanuel F. Mongodin, Amy R. Sapkota

**Affiliations:** 1 Department of Global, Environmental, and Occupational Health, School of Public Health, University of Maryland, College Park, MD USA; 2 Center for Health Equity, School of Public Health, University of Maryland, College Park, MD USA; 3 Department of Epidemiology and Biostatistics, School of Public Health, University of Maryland, College Park, MD USA; 4 Department of Microbiology and Immunology, Institute for Genome Sciences, University of Maryland School of Medicine, Baltimore, MD USA; 5 Present Address: Division of Lung Diseases, National Institutes of Health (NIH), National Heart, Lung and Blood Institute (NHLBI), Bethesda, MD USA

**Keywords:** Microbiology, Applied microbiology, Clinical microbiology, Environmental microbiology, Policy and public health in microbiology

## Abstract

Tobacco use significantly influences the oral microbiome. However, less is known about how different tobacco products specifically impact the oral microbiome over time. To address this knowledge gap, we characterized the oral microbiome of cigarette users, smokeless tobacco users, and non-users over 4 months (four time points). Buccal swab and saliva samples (n = 611) were collected from 85 participants. DNA was extracted from all samples and sequencing was carried out on an Illumina MiSeq, targeting the V3–V4 region of the 16S rRNA gene. Cigarette and smokeless tobacco users had more diverse oral bacterial communities, including a higher relative abundance of *Firmicutes* and a lower relative abundance of *Proteobacteria*, when compared to non-users. Non-users had a higher relative abundance of *Actinomyces, Granulicatella, Haemophilus, Neisseria, Oribacterium, Prevotella, Pseudomonas, Rothia*, and *Veillonella* in buccal swab samples, compared to tobacco users. While the most abundant bacterial genera were relatively constant over time, some species demonstrated significant shifts in relative abundance between the first and last time points. In addition, some opportunistic pathogens were detected among tobacco users including *Neisseria subflava, Bulleidia moorei* and *Porphyromonas endodontalis*. Overall, our results provide a more holistic understanding of the structure of oral bacterial communities in tobacco users compared to non-users.

## Introduction

Over 700 bacterial species are known to inhabit the oral cavity, collectively known as the oral microbiome^1,2^. It is one of the most diverse microbial communities in the human body and studies over the past two decades have characterized these communities in great depth^2,3^. Given a normal body temperature of 37 °C and optimal nutrients, the oral cavity provides a stable environment for a variety of bacterial species to survive in their specific niches^4^. These different niches or surfaces in the oral cavity comprise either hard (e.g., teeth, palates, gingival sulcus) or soft tissues (e.g., tongue, cheeks)^2^. Specific bacteria preferentially colonize these different surfaces due to variable surface adhesins and oral receptors^5^. For instance, the microbiome of saliva is more similar to that of the tongue, while the microbiome of soft tissues is largely dissimilar from that of hard surfaces like the teeth^6^. Nevertheless, results from previous oral microbiome studies are difficult to compare given the variability in sampling methods and sampling sites within the oral cavity^7–11^.

Previous studies have decisively demonstrated, however, that one of the most significant factors influencing the composition of the oral microbiome is tobacco use^8,10,12^. Chemical constituents and bacterial communities from tobacco products and smoke heavily influence the oral microbiome of tobacco users^10,12–15^, causing shifts from eubiosis to dysbiosis. While a change in bacterial community composition within a microenvironment is generally considered dysbiosis^16^, oral dysbiosis is specifically characterized by a loss of beneficial bacteria along with a potential increase in pathogenic bacteria^17^. Multiple factors related to smoking and smokeless tobacco use affect oral microbial homeostasis. For example, the use of smokeless tobacco has been shown to be associated with a depletion of beneficial bacterial genera such as *Lactobacillus* and *Haemophilus* in the oral cavity when compared to non-users^15^. Similarly, cigarette smoking has been shown to reduce the diversity of oral Gram-positive bacterial populations when compared to non-users^18,19^, and the duration of cigarette smoking has been shown to affect the prevalence of pathogenic bacteria in the oral cavity^20^.

However, less is known about how the use of specific tobacco products (e.g., cigarettes vs. smokeless tobacco) may result in different changes in the human oral microbiome over time. These changes are important because they could result in adverse health impacts among users. The oral microbiome is closely linked to the physiological state of the body, especially with regard to changes in the immune system^21–23^. These changes can potentially lead to substantial shifts in the symbiotic balance between host and microbiome, as well as pathogen colonization, leaving individuals susceptible to disease development^21,22,24^. For example, a shift from Gram-positive aerobes to Gram-negative anaerobes in the oral cavity has been linked to the development of periodontal disease^25^. Therefore, studying the temporal variability of oral bacterial communities is useful in understanding the role of these communities in both disease development and the maintenance of a healthy mouth^26^. To improve our understanding of the effects of specific tobacco product use on oral microbiome dysbiosis over time, we employed next-generation sequencing approaches to perform a comprehensive comparison of temporal changes in the oral microbiome of cigarette smokers, smokeless tobacco users, and non-users over 4 months.

## Results

### Study participants

A total of 85 (24 CG, 18 ST, and 43 NU) participants were included in the study (Table 1). The majority of the participants were male and single (never married) (Table 1). 7%, 33% and 55% of all participants were Asian, Black and White, respectively (Table 1). The majority of the participants were employed full time and had greater than 12 years of formal education (Table 1).

**Table 1:** Cigarette users (CG), smokeless tobacco users (ST) and non-users (NU) included in this study.

	CG (n = 24)	ST (n = 18)	NU (n = 43)
	*n(%)*	*n(%)*	*n(%)*
Age, mean (SD)	35.70 (12.56)	28.55 (6.31)	31.02 (8.51)
Sex			
Male	17 (70.83)	17 (94.44)	35 (81.40)
Female	7 (29.17)	1 (5.56)	8 (18.60)
Marital status			
Legally married	0	0	4 (9.30)
Living with partner/cohabiting	1 (4.17)	0	5 (11.63)
Single, never married	19 (79.17)	18 (100)	33 (76.74)
Divorced	3 (12.50)	0	1 (2.33)
Separated	1 (4.17)	0	0
Ethnicity			
Hispanic/Latino	4 (16.67)	0	1 (2.33)
Non Hispanic/Latino	18 (75.0)	17 (94.44)	42 (97.67)
Refused	2 (8.33)	1 (5.56)	0
Race			
Asian	2 (8.33)	1 (5.56)	3 (6.98)
Black or African-American	14 (58.33)	1 (5.56)	13 (30.23)
Mixed	0	0	2 (4.65)
Native Hawaiian or Pacific Islander	0	1 (5.56)	1 (2.33)
White	8 (33.33)	15 (83.33)	24 (55.81)
Employment			
Full time	14 (58.33)	6 (33.33)	28 (65.12)
Military services	0	1 (5.56)	0
Part time-irregular hrs	0	0	1 (2.33)
Part time-regular hrs	3 (12.50)	5 (27.78)	2 (4.65)
Refused	1 (4.17)	0	0
Retired/disabled	0	0	1 (2.33)
Student	3 (12.50)	5 (27.78)	11 (25.58)
Unemployed	3 (12.50)	1 (5.56)	0
Years of formal education			
≤ 12 years	7 (29)	2 (11)	4 (9.3)
> 12 years	17 (71)	16 (89)	38 (88.3)
Refused	0	0	1 (2.32)

The majority of cigarette users smoked 6–10 cigarettes per day and smoked filtered, menthol, and full flavor cigarettes (Table 2). The majority of the smokeless tobacco users used tobacco for 20–30 days in the past month. While Newport was the most popular brand of cigarettes, followed by Marlboro, among the cigarette users, Copenhagen, Grizzley, and Skoal were the most popular brands among the smokeless tobacco users. Seventeen (70.83%) cigarette users, 16 (89.89%) smokeless tobacco users, and 12 (27.91%) non-users had tried smoking a cigar, cigarillo, or little cigar previously. Eighteen (75%) cigarette users, ten (55.5%) smokeless tobacco users, and three (7%) non-users had used electronic cigarettes previously. Our nicotine and cotinine data (Figure S1) validated our tobacco use questionnaire data, demonstrating statistically significantly (*p* < 0.05) lower levels of both cotinine and nicotine concentrations in the saliva of the NU group compared to the CG and ST groups across all time points.

**Table 2:** Tobacco use of cigarette users (CG), smokeless tobacco users (ST) and non-users (NU) included in this study.

	CG (n = 24)	ST (n = 18)	NU (n = 43)
	*n (%)*	*n (%)*	*n (%)*
What brand of cigarette did you smoke most often?			
American Spirits	1 (4.17)	0 (0)	0 (0)
Marlboro	8 (33.33)	2 (11.11)	0 (0)
Newport	11 (45.83)	1 (5.56)	0 (0)
Camel	3 (12.5)	1 (5.56)	0 (0)
Djarum	1 (4.17)	0 (0)	0 (0)
Were they filtered or unfiltered?			
Filtered	22 (91.67)	4 (22.22)	0 (0)
Unfiltered	2 (8.33)	0 (0)	0 (0)
Were they menthol, non-menthol/plain, special or mild, or some other flavor?			
Menthol	15 (62.5)	1 (5.56)	0 (0)
Non-menthol/Plain	7 (29.17)	2 (11.11)	0 (0)
Special/Mild	2 (8.33)	1 (5.56)	0 (0)
Were they full flavor, light, or ultra-light?			
Full flavor	19 (79.177)	3 (16.67)	0 (0)
Ultra light	2 (8.33)	0 (0)	0 (0)
Light	3 (12.5)	1 (5.56)	0 (0)
Were they regular, Kings, 100’s, or 120’s?			
Regular	20 (83.33)	2 (11.11)	0 (0)
100's	2 (8.33)	2 (11.11)	0 (0)
Kings	2 (8.33)	0 (0)	0 (0)
How many cigarettes do you smoke in a normal day?			
< 5	0 (0)	3 (16.67)	0 (0)
6–10	15 (62.5)	1 (5.56)	0 (0)
11–15	5 (20.83)	0 (0)	0 (0)
16–20	3 (12.5)	0 (0)	0 (0)
What brand of smokeless (or spitless) tobacco do you usually use?			
Copenhagen	1 (4.17)	6 (33.33)	0 (0)
Grizzley	0 (0)	5 (27.78)	0 (0)
Skoal	0 (0)	3 (16.67)	0 (0)
Other	0 (0)	4 (22.22)	0 (0)
How many days did you use smokeless tobacco in the past month?			
< 10	24 (100)	1 (5.56)	43 (100)
10–19	0 (0)	4 (22.22)	0 (0)
20–30	0 (0)	13 (72.22)	0 (0)
Have you ever used or tried smoking a cigar, cigarillo, or little cigar?			
Yes	17 (70.83)	16 (88.89)	12 (27.91)
No	7 (29.17)	2 (11.11)	25 (58.14)
Was the product you tried a cigar, cigarillo, or little cigar?			
Cigar	7 (29.17)	12 (66.67)	9 (20.93)
Little cigar	5 (20.83)	1 (5.56)	0 (0)
Cigarillo	5 (20.83)	3 (16.67)	1 (2.33)
Don't know	7 (29.17)	1 (5.56)	27 (62.79)
Have you ever used or tried an electronic cigarette?			
Yes	18 (75)	10 (55.56)	3 (6.98)
No	6 (25)	8 (44.44)	34 (79.07)

### Sequencing data

In order to evaluate oral microbiome changes associated with the use of smokeless tobacco and cigarettes over time, we analyzed 16S rRNA sequencing reads from buccal swabs and saliva samples.

A total of 32,276,329 sequencing reads were obtained from 611 samples, with a mean of 52,898.36 sequences per sample (SD ± 29,608.81). To ensure appropriate sequence coverage across samples, the Good’s estimate of coverage was calculated for each sample, and samples with Good’s values ≤ 0.95 were removed from further downstream analysis. After quality filtering, a total of 32,265,093 reads were obtained from 556 samples for downstream analysis, with a maximum of 167,127 and a minimum of 2301 (average 58,030.74; SD 25,838.92) reads/sample. The total number of sequences from 250 buccal swab samples was 14,636,962 and that from 306 saliva samples was 17,639,367. Overall, sequences were clustered into 6092 OTUs.

### Bacterial diversity and differentially abundant bacterial species across time points

**Figure 1:**
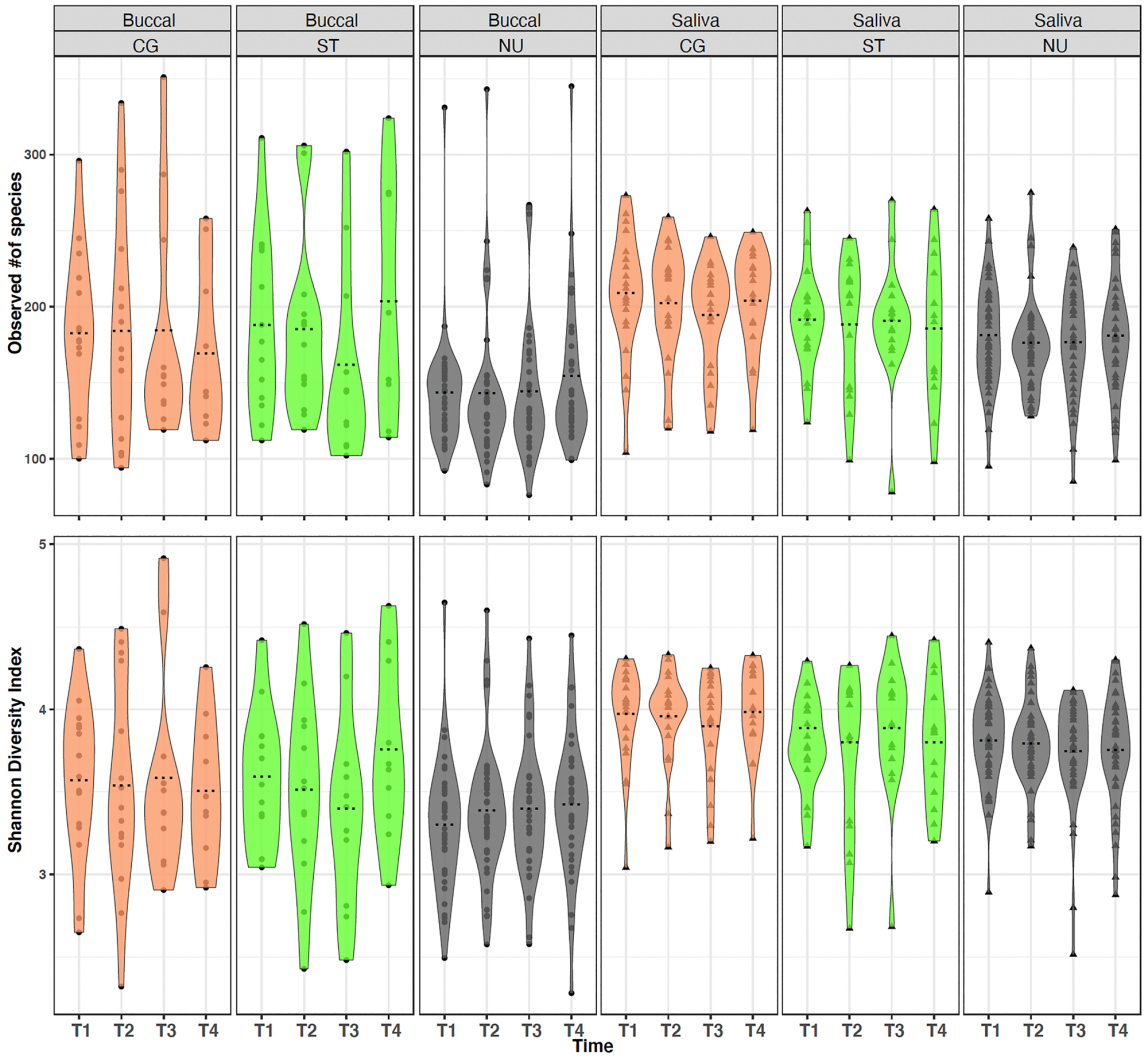
Alpha diversity analysis of buccal swab and saliva samples, by user group, across four-time points. Colors represent the user groups (cigarette user (CG), orange; smokeless tobacco user (ST), green; non-user (NU), grey). Alpha diversity was measured for time-points 1 through 4 (T1–T4) and compared using ANOVA with Tukey’s HSD post hoc test.

Alpha diversity within buccal swab and saliva samples across the four-time points (T1–T4) was calculated using the Observed number of species and the Shannon diversity index (Fig. 1). Looking across the four sampling events, we observed no significant (*p* > 0.05) differences in alpha diversity among buccal swab or saliva samples within any of the user groups. Computing beta diversity on Bray–Curtis distances, our data did not show any significant effect (*p* > 0.05) of time on the bacterial community composition of both buccal swab and saliva samples (Figure S2).

**Figure 2:**
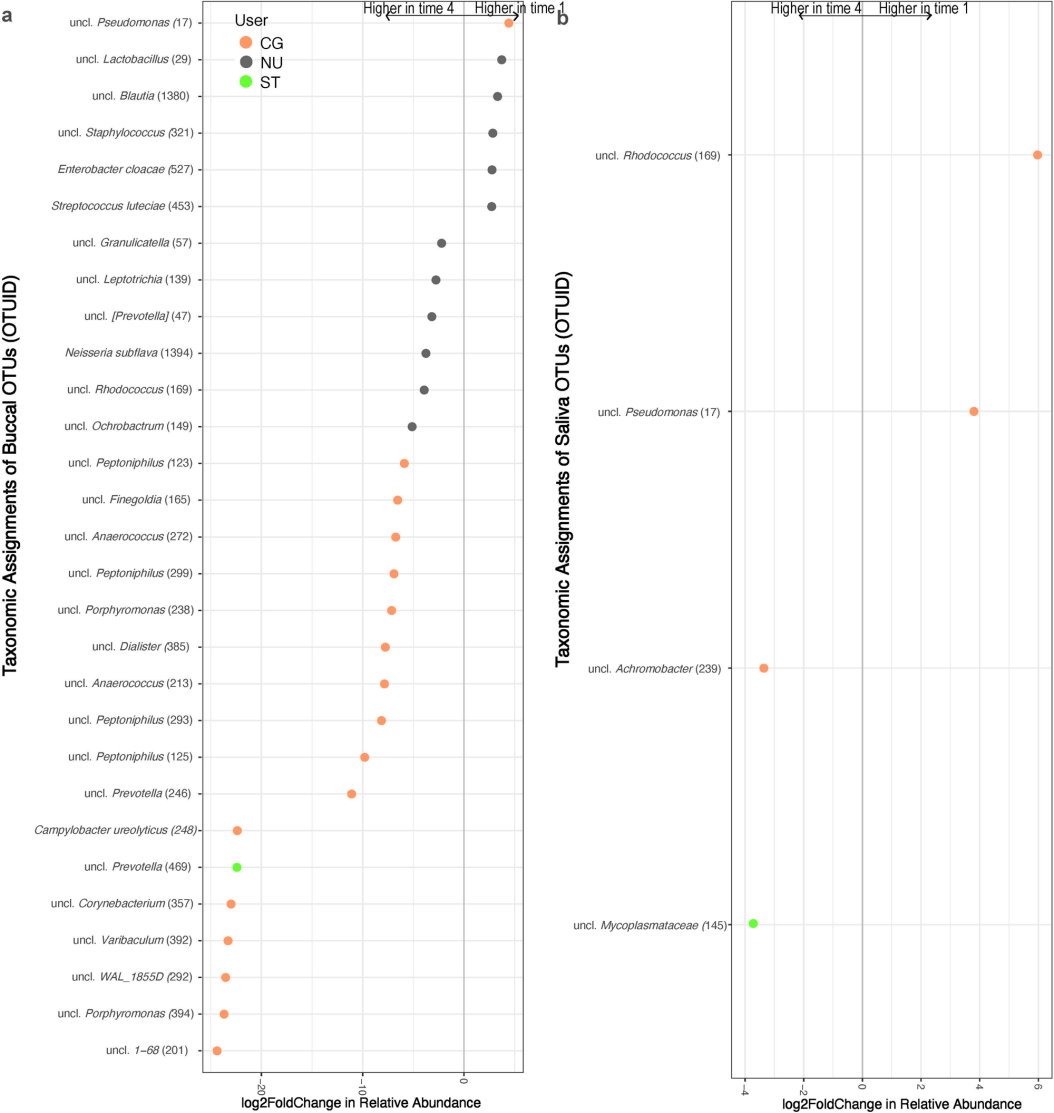
Differential abundance of bacterial OTUs in (**a**) buccal swab and (**b**) saliva samples that were statistically significantly different (α = 0.001) between time-points 1 and 4. The OTUs are colored by user groups (cigarette user (CG), orange; smokeless tobacco user (ST), green; non-user (NU), grey). A positive log2-fold change value denotes an OTU that is significantly higher in time-point 1 samples, while a negative log2-fold change indicates an OTU that is significantly higher in time-point 4 samples. The grey line and arrows highlight the conversion in log2-fold change from negative to positive values.

Moreover, there were no significant temporal differences observed among the dominant six bacterial genera in buccal swab or saliva samples within any of the user groups over the four time points (Figure S3). However, at the bacterial OTU level, we observed statistically significant (*p* < 0.05) differential abundance shifts in OTUs in both buccal and saliva samples across the three user groups between T1 and T4 (Fig. 2). Within the buccal swab samples, the majority of the OTUs that were at a statistically significantly (*p* < 0.05) higher relative abundance at T4 (16 OTUs) belonged to the CG group, while six OTUs were in the NU group and only one OTU was in the ST group (Fig. 2a). In contrast, only 5 OTUs in the NU group were at a statistically significantly higher relative abundance at T1 compared to T4, and only one OTU in the CG group was at a significantly higher relative abundance at T1 compared to T4.

Within the saliva samples, comparing across the T1 and T4 time points, two OTUs in CG samples were at a statistically significantly higher relative abundance at T1, while one OTU in CG samples was at a higher relative abundance at T4 (Fig. 2b). One OTU from ST samples was at a statistically significantly higher relative abundance at T4 compared to T1. There were no OTUs in NU saliva samples that were at a statistically significantly different relative abundance (*p* > 0.05) between T1 and T4.

### Bacterial community diversity between user groups

**Figure 3:**
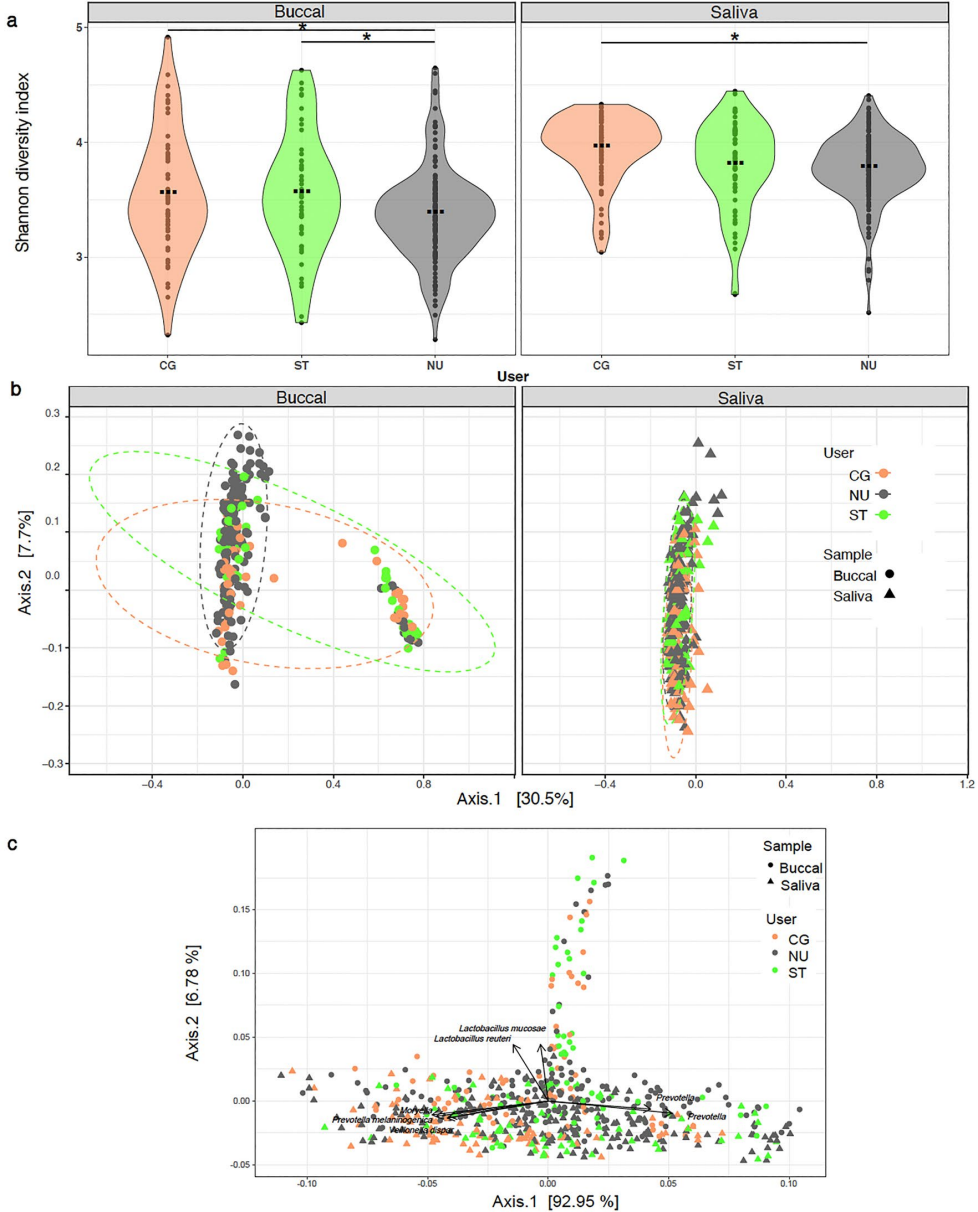
Bacterial diversity of buccal swab samples and saliva samples from cigarette users (CG), smokeless tobacco users (ST), and non-users (NU). (**a**) Alpha diversity violin plots were generated using the Shannon diversity index. Black lines represent significant changes between user groups and * represents statistically significant differences identified through Tukey’s HSD *posthoc* test (*p* < 0.05). (**b**) Beta diversity was visualized through PCoA plots of Bray–Curtis computed distances among sample types. Ellipses are drawn at 95% confidence intervals. (**c**) PCoA bi-plot of beta diversity based on Bray–Curtis computed distances of buccal swab and saliva samples collected from cigarette users (CG), smokeless tobacco users (ST), and non-users (NU) showing significant correlations with specific bacterial taxa. The black arrows reflect the relationships between the bacterial taxa, with the direction of the point of the arrow showing increasing values of the taxa, and the cosine of the angle between the arrows reflecting the correlations between the taxa.

Since there was no statistically significant effect of time on the alpha diversity indices, the four-time points for each participant were merged in downstream analyses. Across all user groups, alpha diversity, measured using the Shannon diversity index, was significantly lower in buccal swab samples (average 3.44 (± 0.46 SD)) (*p* < 0.05) compared to saliva samples (average 3.81 ± 0.32) across all user groups (Fig. 3a). Comparing alpha diversity in buccal swab samples between the three groups, the NU group was characterized by the lowest Shannon diversity index (average 3.36 ± 0.40). The Shannon diversity indices among buccal swab samples for the CG (average 3.55 ± 0.52) and ST (average 3.56 ± 0.52) groups were comparable. Similar to the buccal swab samples, Shannon diversity among the saliva samples in the NU group was also found to be the lowest (average 3.76 ± 0.30) compared to the CG (average 3.94 ± 0.29) and ST (average 3.80 ± 0.38) groups.

To evaluate the effects of tobacco use on bacterial community composition, we computed beta diversity on Bray–Curtis distances and performed a PCoA analysis. Within the buccal swab samples, 9% of the variation in the bacterial community composition was explained by user groups (CG, ST, and NU) (Adonis R: 0.02, *p* < 0.001) (Fig. 3b). However, this difference in beta diversity was not found when saliva samples were compared between the three groups. Constructing a biplot of bacterial species on the PCoA plot demonstrated that the smaller cluster of buccal swab samples was potentially being driven by *Lactobacillus mucosae* and *L. reuteri* (Fig. 3c).

### Diversity and composition of bacterial communities across race and gender

Beta diversity on Bray–Curtis dissimilarity distances was computed by gender and race across all participants and their samples. 4% of the variation in bacterial community composition was significantly explained by race (Figure S4). Comparing bacterial genera, the buccal swab samples of tobacco users (cigarette and smokeless tobacco) had a higher relative abundance of *Veillonella* among Asian participants compared to other races, while this was not observed among the non-users (Figure S5). Comparing within the buccal swab samples, while *Streptococcus* and *Rothia* were at a lower relative abundance in all white participants (CG, ST, and NU), *Neisseria* and *Haemophilus* were at a lower relative abundance in white tobacco users (CG and ST) when compared with that from Black participants. *Lactobacillus* and *Prevotella* were at a higher relative abundance in buccal swabs from white tobacco users when compared to that from Black participants. Within the saliva samples from smokeless tobacco users, the highest relative abundance of *Streptococcus* was observed among Asians, and that of *Veillonella* and *Prevotella* among mixed-race participants when compared to other races.

Comparing genders, 9% of the variation in bacterial community composition was explained by gender (Figure S6). Across both genders, *Streptococcus* was at the highest relative abundance in all samples (Figure S7). Within the buccal swab samples, the top five bacterial species (*Streptococcus, Veillonella, Prevotella, Rothia* and *Actinomyces*) were higher in relative abundance in males across tobacco users (cigarette and smokeless tobacco) when compared to females of the same user group. Within the non-users, the above-mentioned genera showed a similar trend in males except for *Prevotella* and *Actinomyces* which were at similar average relative abundances between males and females.

Within the saliva samples from females, *Streptococcus, Rothia* and *Granulicatella* were at a higher relative abundance when compared to that from males. *Prevotella* and *Neisseria* were observed to be at a higher relative abundance among male tobacco users when compared to female tobacco users.

### Relative abundance of bacterial communities between user groups

The top five bacterial phyla identified in all samples belonged to *Firmicutes, Bacteroidetes, Actinobacteria, Proteobacteria and Fusobacteria* (Figure S8). Comparing across buccal swabs from the three user groups, the relative abundance of *Firmicutes* was the lowest in the NU group (52%), compared to the CG (63.1%) and ST (63.1%) groups, while the relative abundance of *Proteobacteria* was highest in the NU group (16%) when compared to the CG (9%) and ST (12.5%) groups. Similar to the buccal swab samples, saliva samples from the NU group also had the lowest relative abundance of *Firmicutes* (44%) (CG (47%) and ST (46%)) and the highest relative abundance of *Proteobacteria* (14%) (CG (8.4%) and ST (12%)). All three groups were characterized by a similar relative abundance of *Bacteroidetes*, *Fusobacteria* and *Actinobacteria.*

**Figure 4:**
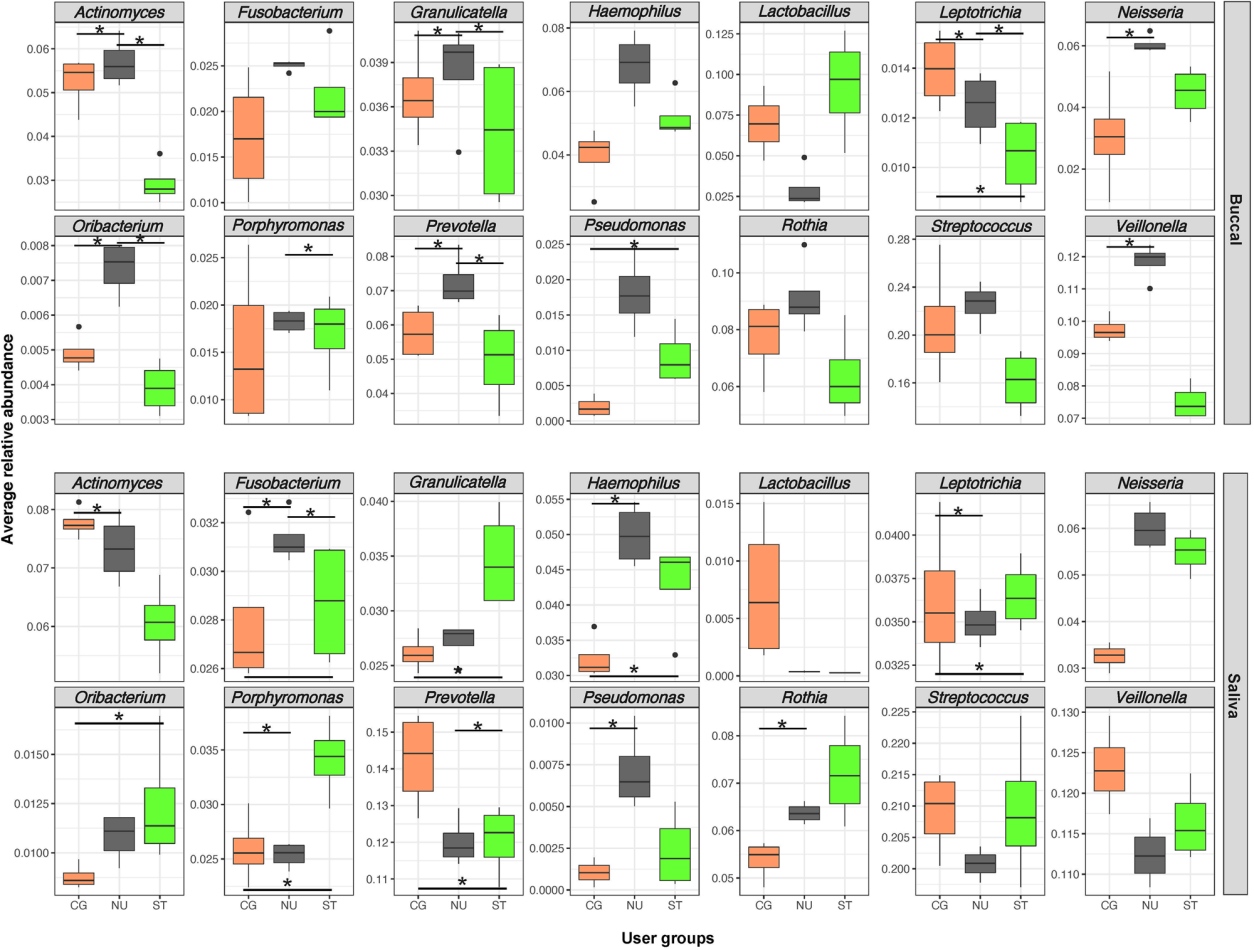
Box plots of relative abundance of the top 14 bacterial genera by user group: cigarette user (CG, orange), smokeless tobacco user (ST, green), and non-user (NU). (*) represents *p*-value < 0.05.

Comparing the relative abundance of bacterial genera within buccal swab samples across user groups, *Actinomyces, Granulicatella, Leptotrichia, Prevotella* and *Oribacterium* were at statistically significantly different relative abundances in the NU group compared to either the CG or ST groups (Fig. 4). Among the above-mentioned five bacterial genera, while the highest relative abundance of *Leptotrichia* was among the CG group, the other four genera (*Actinomyces, Granulicatella, Prevotella*, and *Oribacterium*) were at a higher relative abundance in the NU group. In the buccal swab samples, the relative abundance of *Neisseria* and *Veillonella* was significantly higher in the NU group compared to the CG group. Comparing the relative abundance of bacterial genera in buccal swabs from the ST and CG groups, the relative abundance of *Leptotrichia* was significantly lower and *Pseudomonas* was significantly higher in the ST group compared to the CG group. The buccal swabs of the ST group also had the lowest relative abundance of *Actinomyces* and *Veillonella* compared to the other user groups. Buccal swab samples had a higher relative abundance of *Granulicatella, Haemophilus, Lactobacillus* and *Pseudomonas* compared to saliva samples across all participant groups (Figure S9).

Within the saliva samples, the relative abundance of *Fusobacterium, Haemophilus, Pseudomonas* and *Rothia* was statistically significantly higher in the NU group compared to the CG group (Fig. 4). Relative abundances of *Fusobacterium*, *Granulicatella, Haemophilus, Leptotrichia, Neisseria, Oribacterium, Porphyromonas,* and *Rothia* were higher in the saliva samples of the ST group compared to the CG group. *Actinomyces, Lactobacillus, Prevotella,* and *Veillonella* were at a lower relative abundance in the saliva samples of the ST group compared to the CG group. Finally, saliva samples had a higher relative abundance of *Actinomyces, Fusobacterium, Leptotrichia, Oribacterium, Porphyromonas,* and *Prevotella* compared to buccal swab samples across all participant groups (Figure S9).

### Differentially abundant bacterial genera across user groups

**Figure 5:**
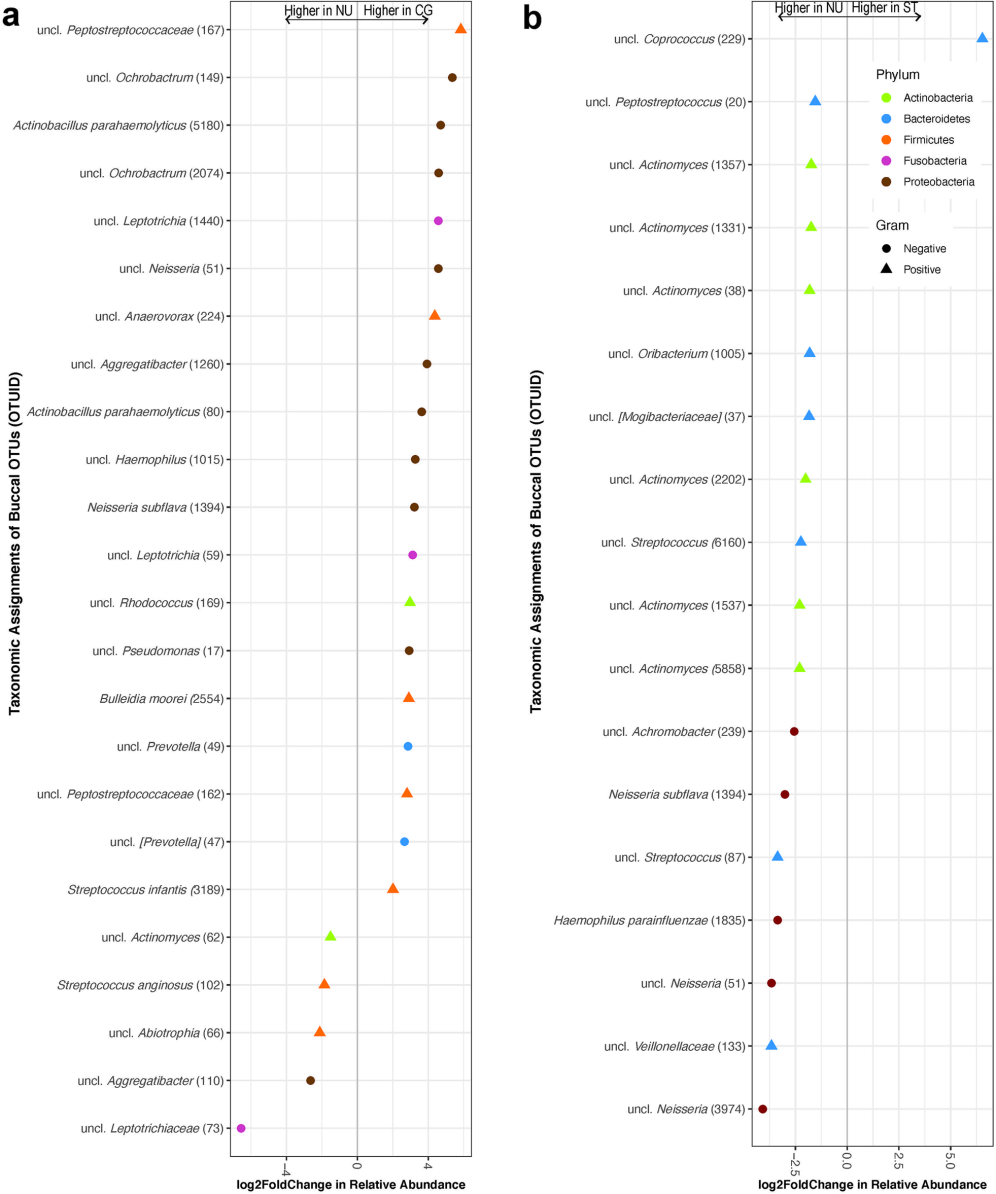
Relative abundance of bacterial OTUs in buccal swab samples that were statistically significantly different (α = 0.001) between (**a**) non-users (NU) and cigarette users (CG) and (**b**) non-users (NU) and smokeless tobacco users (ST). The OTUs are colored by their bacterial phyla. Circles represent Gram-negative and triangles represent Gram-positive bacteria. A positive log2-fold change value denotes an OTU that is significantly higher in user (CG or ST) samples, while a negative log2-fold change indicates an OTU that is significantly higher in NU samples. The grey line and arrows highlight the conversion in log2-fold change from negative to positive values.

In terms of statistically significantly different (*p* < 0.05) OTUs within buccal swab samples across the three user groups, three Gram-positive OTUs (*Streptococcus anginosus* (OTU#102), *Actinomyces* (OTU#62) and *Abiotrophia* (OTU#66)), and two Gram-negative OTUs (*Aggregatibacter* (OTU#110) and *Leptotrichiaceae* (OTU#73)) were at a statistically significantly higher relative abundance in the NU group compared to the CG group (Fig. 5a). 19 OTUs (six Gram-positive and 13 Gram-negative) were at a statistically significantly higher relative abundance in the CG group compared to the NU group. 17 OTUs (five Gram-negative and 12 Gram-positive) were at a statistically significantly higher relative abundance in the NU group compared to the ST group, while only one Gram-positive bacteria (*Coprococcus* (OTU#229)) was at a higher relative abundance in the ST group compared to the NU group (Fig. 5b).

**Figure 6:**
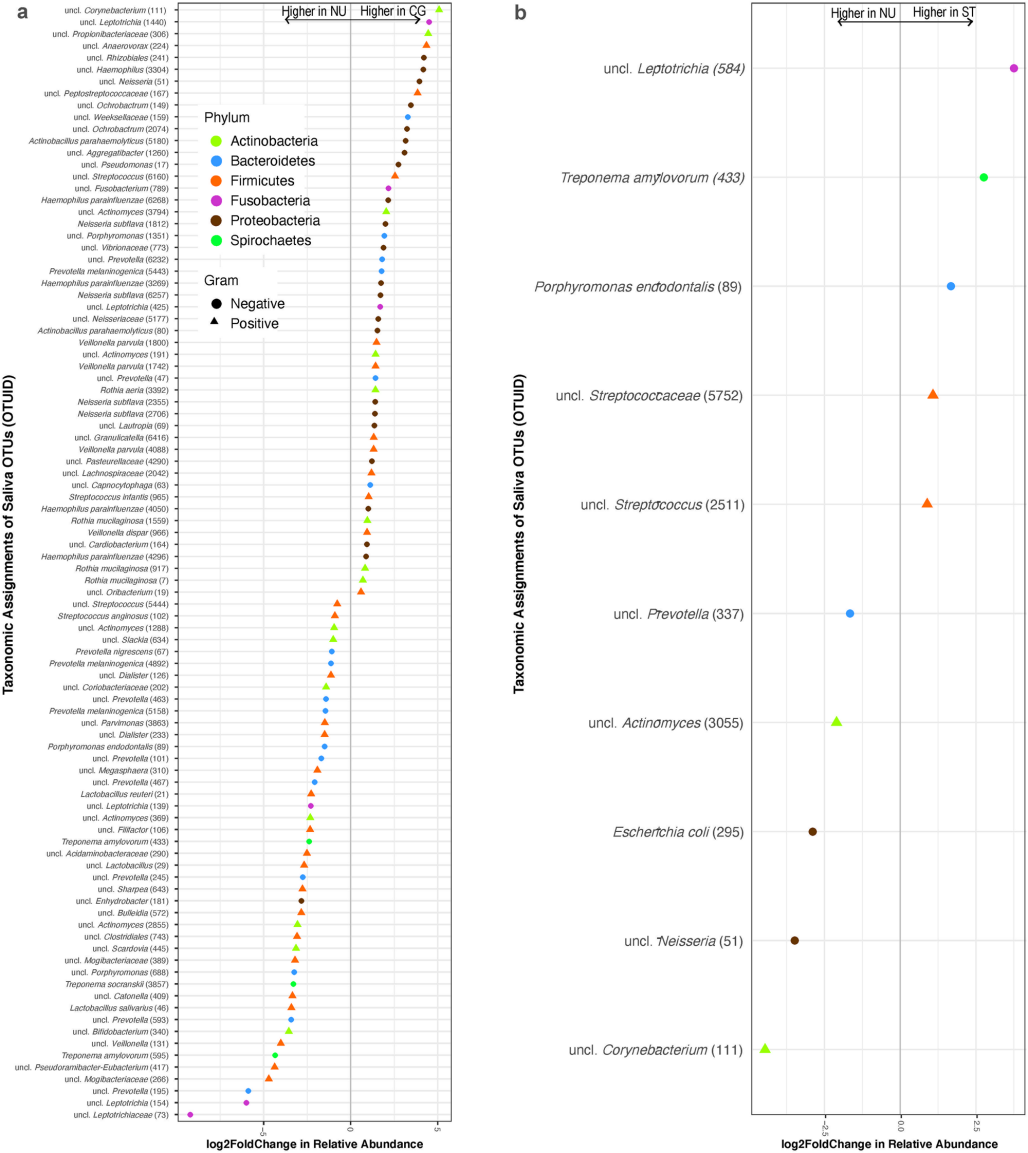
Relative abundance of bacterial OTUs in saliva samples that was statistically significantly different (α = 0.001) between (**a**) non-users (NU) and cigarette users (CG) and (**b**) non-users (NU) and smokeless tobacco users (ST). The OTUs are colored by their bacterial phyla. Circles represent Gram-negative and triangles represent Gram-positive bacteria. A positive log2-fold change value denotes an OTU that is significantly higher in user (CG or ST) samples, while a negative log2-fold change indicates an OTU that is significantly higher in NU samples. The grey line and arrows highlight the conversion in log2-fold change from negative to positive values.

Comparing bacterial OTUs in saliva samples, 18 Gram-negative and 26 Gram-positive OTUs were at a statistically significantly higher relative abundance in the CG group compared to the NU group, while 31 Gram-negative and 19 Gram-positive OTUs were at a statistically significantly higher relative abundance in the NU group compared to the CG group (Fig. 6a). Comparing among the NU and ST groups, 2 Gram-positive and 3 Gram-negative OTUs were at a statistically significantly higher relative abundance in the ST group (Fig. 6b**)**.

## Discussion

Cigarettes and smokeless tobacco products contain multiple chemical and microbiological constituents^27,28^ that can alter a user’s oral microbiome^8,10,12–15^. In this study, we found that using smokeless tobacco or smoking cigarettes played a distinct role in dictating the changes in the oral microbiome of the user. Furthermore, while the majority of the oral microbiome composition remained relatively stable over time for individual tobacco users, the relative abundance of a few oral bacteria changed significantly over the 4-month study period.

While previous studies evaluating the impacts of smoking on the oral microbiome have either categorized the smokers as light/heavy smokers or short/mild/long-term smokers based on their reported cigarette consumption (per/day, pack/year, or nicotine dependence levels)^7,29^, no previous studies, to our knowledge, have followed the participants over time to evaluate the stability of the oral microbiome among tobacco users compared to non-users. Here, in our longitudinal study, interestingly, we did not observe any significant effect of time on the bacterial diversity of buccal swab and saliva samples from all three user groups (Fig. 1; Figure S2). Previous oral microbiome studies reported from the Human Microbiome Project demonstrated temporal stability of the heterogeneous oral microbiome in healthy non-smokers^30^. In addition, salivary microbiome diversity has been found to remain relatively stable within individuals over a short-time period^31–34^ up to a year^35^, and this result did not change with the use of antibiotics^36,37^. In another study, after adjusting for the use of antibiotics, estimates of temporal oral microbiome stability did not significantly change^38^. Nevertheless, even though the overall diversity and composition of the oral microbiome remains relatively stable over time, comparing between our T1 and T4 time points, our data demonstrated significant changes in the differential abundance of a few bacterial species across tobacco users (CG and ST) and non-users (Fig. 2).

Even though we provide evidence of temporal stability of the healthy oral microbiome, environmental perturbations, like smoking tobacco, significantly affect oral bacterial community diversity and composition^7–10^. In multiple body sites, such as the gut, dysbiosis is characterized by a loss of bacterial diversity^39^. However, in the mouth, an increase in bacterial diversity often characterizes dysbiosis^40^. Here, we found that the buccal swabs and saliva of non-users had significantly lower alpha diversity when compared to tobacco users (CG or ST) (Fig. 3). These findings corroborate previous studies that have found higher levels of bacterial diversity within the oral microbiomes of tobacco users compared to non-users^15,41,42^. From a disease development perspective, interestingly, the initiation and perpetuation of periodontal diseases has been associated with an increase in bacterial species diversity rather than a decrease^43,44^. Moreover, smokers have been characterized as harboring an oral microenvironment that potentially supports early colonization and enrichment of bacterial pathogens compared to non-smokers^41,45,46^. Subsequently, the abundance of pathogenic bacterial species in smokers’ subgingival microbiomes has been linked to loss of resiliency and decreased resistance to future episodes of gingivitis and periodontitis^44,47^.

While previous studies have revealed changes in diversity in the oral bacterial microbiome with tobacco use, these changes are not consistent across all sampling sites within the oral cavity^10^. Here, we identified that the majority of buccal swab and saliva samples were characterized by the presence of *Streptococcus, Veillonella, Prevotella, Rothia, Actinomyces*, *Haemophilus,* and *Neisseria*. However, the relative abundances of these and other bacteria changed by sample type across all three user groups (CG, ST, and NU) (Fig. 4). For example, in comparison to tobacco users (CG and ST), non-users had a higher relative abundance of *Actinomyces, Granulicatella, Haemophilus, Neisseria, Oribacterium, Prevotella, Pseudomonas, Rothia*, and *Veillonella* in buccal swab samples. However, in the saliva samples, while *Haemophilus, Neisseria,* and *Pseudomonas* were also at a higher relative abundance in non-users, the other above-mentioned genera did not follow the same patterns observed in the buccal swab samples. Significant differences in bacterial genera between the two oral sampling sites (buccal swabs vs. saliva) point towards distinct microbial niches, as shown previously in studies comparing the buccal mucosa, saliva, dental plaques, palates, and the tongue^10,30^. The differences in proportions of bacterial taxa in these different oral sites might be due to different site receptors on bacterial cell walls, specific species interactions, and specific surface properties that affect the bacteria’s survival and growth^48^.

Previous studies also have established that smoking causes oral microbiome dysbiosis with concurrent enrichment of pathogens and depletion of commensals^12,41,49^. Furthermore, culture-based studies have shown that tobacco smoking inhibits the growth and reduces the diversity of Gram-positive bacteria (encompassing multiple pathogenic species) when compared to Gram-negative bacteria^19,50^. Consistent with previous findings, we identified statistically significantly lower differentially abundant Gram-positive species in the saliva from cigarette users compared to that from non-users (Fig. 4; Figure S9). Evaluating the relative abundances of bacterial genera in buccal swab samples, we identified a number of Gram-positive genera, such as *Actinomyces, Granulicatella, Oribacterium, Rothia*, and *Streptococcus,* that were at a lower relative abundance in cigarette smokers compared to non-users (Fig. 4; Figure S9). In the saliva samples, similar trends were seen except with regard to *Actinomyces* and *Streptococcus*. Comparing the smokeless tobacco users to non-users, we found similar trends for the above-mentioned Gram-positive genera in the buccal swab samples. However, in the saliva samples, this was not true for *Granulicatella, Oribacterium, Rothia*, and *Streptococcus*. This difference between cigarette smokers and smokeless tobacco users might be due to the fact that smokeless tobacco is not burned and inhaled as in cigarette smoking, which deposits toxicants from cigarette smoke, depleting oxygen and increasing salivary pH^8^. Recent studies have demonstrated a higher relative abundance of genera such as *Rothia, Lactobacillus,* and *Streptococcus* in the oral microbiome of smokeless tobacco users compared to non-users^51^. Gram-positive *Rothia* is a usual member of the oral microbiome, but is more abundant in individuals with tongue leukoplakia lesions and periodontal diseases compared to healthy controls^52–54^. This genus is also known to produce high levels of acetaldehyde while lacking acetaldehyde dehydrogenases to detoxify acetyl aldehyde (a compound that can contribute to oral and gastrointestinal carcinogenesis)^55^.

In addition to the Gram-positive species that were differentially abundant in the oral microbiome between tobacco user groups, a few of the Gram-negative species were also characterized by notable changes in relative abundance across user groups. One of the most abundant commensals and early colonizers of the oral cavity, *Neisseria,* was shown to be depleted in the buccal swabs and saliva of tobacco users (CG and ST) compared to that of non-users. This was consistent with previous studies that found *Neisseria* (a Gram-negative member of the *Proteobacteria*) to be depleted in smokers’ oral mucosas^56^, lower respiratory tracts^57^, nasopharynges, and oropharynges^58^. Usually considered obligate aerobes, both *Neisseria* and *Rothia* can also thrive in anaerobic biofilms and exhibit active denitrification^59,60^, reducing nitrate to nitrite and eventually nitric oxide, a free radical with antimicrobial properties. Nitrite molecules can react with various tobacco alkaloids to generate carcinogenic tobacco-specific nitrosamines (TSNAs). Therefore, depletion of *Neisseria* and *Rothia* species in tobacco users’ oral microbiomes might potentially build an oral ecosystem conducive to producing TSNAs. Comparing the tobacco users (CG and ST), our data also showed a higher relative abundance of *Neisseria* in smokeless tobacco users compared to cigarette users. This might be due to the fact that cigarette smoking is known to increase the acidity of saliva^19^, and *Neisseria* is sensitive to acidic conditions.

While different constituents in tobacco products (cigarettes and smokeless tobacco) might have an inhibitory effect on the relative abundance of several bacterial species^61,62^, smoking has also been linked with an increase in certain oral bacterial genera. Here, we found that the saliva from cigarette users had a higher relative abundance of *Prevotella* (a facultative anaerobe) and *Veillonella* (an obligate anaerobe). *Prevotella* is a dominant member of the gut microbiome but its higher abundance in the gut has also been linked to colon cancer and colitis susceptibility^63–66^. A pilot study demonstrated a significantly higher relative abundance of *Prevotella* in the gut of tobacco smokers^14^, and another study noted an increase in the relative abundance of *P. bivia* and *V. dispar* in the oral microbiome of heavy smokers^7^. We also found a statistically significantly higher relative abundance of *Veillonella* in the buccal swabs of non-users when compared to cigarette users. Commensal *Veillonella* can utilize lactic acid and convert it to weaker acids and in turn produce nitrite from nitrate^67^. With antimicrobial properties, nitrite has been shown to inhibit the growth and metabolism of oral pathogenic bacteria^68^.

There are multiple strengths of this study. First of all, our study included a detailed comparison of the oral microbiome dysbiosis (in both diversity and composition) that occurs among cigarette smokers versus smokeless tobacco users. There are limited studies with a comparable approach that have evaluated changes in the oral microbiome associated with variable tobacco use. Next, by following the participants over 4 months, we evaluated changes in the oral microbiome that might occur with tobacco use over time. Previous studies evaluating the impacts of tobacco use on the oral microbiome have either included only one type of tobacco product user, solely relied on culture-based techniques, or were limited with regard to longitudinal data^8,10,11,14,29,44,47,69–72^. Finally, our total number of samples was robust and participant groups included controls (non-users) matched by age, sex, and race with each of the tobacco users.

Although our study had multiple strengths, there are limitations to note as well. First of all, because we carried out 16S rRNA gene sequencing instead of metagenomic sequencing, we could not explore the functional attributes of the identified oral bacterial communities. In addition, even though we included 85 participants, the majority of our subjects were Black or White. Thus, while we identified significant differences in the oral microbiome across user groups, these results may not be generalizable to larger, more diverse populations. A third limitation to note is that the oral health data that we obtained was self-reported and no oral examinations were performed.

In summary, we observed oral microbiome differences associated with tobacco use, and conclude that dysbiosis of oral bacterial communities is related to the specific type of tobacco product used (e.g., cigarettes vs. smokeless tobacco). Since the use of differing types of tobacco alters the oral microbiome^8,12,19,41,42^, and perturbations in the oral microbiome have been linked to multiple oral and non-oral diseases^73,74^, these altered microbiomes may well play a role in the development of carcinogenic and/or non-carcinogenic outcomes among tobacco users^4,63,75,76^.

## Methods

### Study population

Convenience sampling was conducted in and around the University of Maryland, College Park, MD to recruit participants. Specifically, participants were recruited through word-of-mouth, physical advertisements posted in and around the University of Maryland, and digital advertisements posted through email, campus websites, and social networking sites. To ensure the inclusion of racially diverse participants, we also partnered with the University of Maryland Center for Health Equity and recruited through their existing partnerships with black barbershops in the area.

Subjects were all healthy individuals, 18–55 years of age, who were in good oral health. Exclusion criteria included the use of antibiotics in the last 6 months, dependence on alcohol, diagnosis of pneumonia in the last 6 months, heart or lung problems in the last 6 months, or a diagnosis of emphysema, cancer, hepatitis B virus, hepatitis C virus or HIV. Exclusion criteria also included a diagnosis of dry mouth, untreated cavitated carious lesions, oral abscesses, precancerous or cancerous oral lesions, oral candidiasis, or clinically meaningful halitosis. Participants who had more than 8 missing teeth, had any major dental or oral surgery in the past 6 months, had taken any of the listed drugs within the last 6 months (e.g., systemic antibiotics, antifungals, antivirals, or antiparasitics; oral, intravenous, intramuscular, nasal, or inhaled corticosteroids; cytokines; methotrexate or immunosuppressive cytotoxic agents; or large doses of commercial probiotics) or were pregnant, breastfeeding or planning on becoming pregnant within the next 6 months were also excluded from the study.

Once a potential participant expressed interest in the study, a phone screen was first conducted to ensure that the participant met eligibility criteria and fell into one of two study groups: cigarette users (CG) or smokeless tobacco users (ST). After the recruitment of tobacco users, non-tobacco users (NU) were recruited similarly and matched to each tobacco user participant by age (± 3 years), race, and gender. All cigarette users had smoked more than six cigarettes on a typical day for the previous 3 years and all smokeless tobacco users had used tobacco at least one time per week for the previous whole year. Non-tobacco users either had never used tobacco products or had smoked less than 20 cigarettes or used smokeless tobacco less than 20 times in their lifetime. After sample size calculations (details provided in Supplementary material) 24 participants were required to be enrolled in each user group.

Upon enrollment, participants completed three baseline questionnaires (described below). In addition, we obtained buccal swab and saliva samples from participants once every 30 (± 2) days for four consecutive months (T1–T4) (described in detail below). Participants refrained from using any tobacco products or ingesting food, water, or caffeine for at least two hours prior to sample collection. Participants also refrained from ingesting alcohol for at least 24 h before sample collection. All participants were provided with overall project goals and engaged in the informed consent process. All protocols were approved by the Institutional Review Board (IRB) of the University of Maryland (UMD), College Park. All methods were performed in accordance with the relevant guidelines and regulations set by UMD IRB.

### Baseline questionnaires

Three baseline self-administered questionnaires were completed by each participant: a demographics questionnaire; an oral health and hygiene questionnaire, and a tobacco use questionnaire. The oral health and hygiene questionnaire included questions related to factors that might affect the oral microbiome (e.g., alcohol use, recent dental work, recent upper respiratory and gastrointestinal infections). Questions on the tobacco use questionnaire focused on self-reported measures of tobacco exposure (e.g., brand and type of tobacco used/smoked, amount and frequency of each product used/smoked, use of other types of tobacco (e.g., cigars, little cigars, and electronic cigarettes) and pulmonary history.

### Saliva and buccal swab collection

To obtain saliva samples, participants were asked to let saliva collect in the mouth for at least one minute, and then expel 5 mL of saliva into a labeled 50 mL Falcon tube. 8 mL of RNALater solution (Thermo Fisher, MA) was added to the 50 mL Falcon tube, vortexed, and incubated at 4 °C for 24 h. After incubation, all samples were stored at − 80 °C until DNA extraction. Buccal swabs were collected using four E-swabs (Copan, CA) from four sites inside the oral cavity: the tongue dorsum, the hard palate, and the left and right buccal mucosa. Using the first e-swab, 1 cm^2^ of the center of the tongue was swabbed vigorously for 60 s and the swab was placed in a 50 mL Falcon tube containing 5 mL of RNALater solution. The second e-swab was then used to swab the entire hard palate vigorously for 60 s and the swab was placed in the same 50 mL Falcon tube. The third and fourth e-swabs were then used to swab the left and right buccal mucosa for 60 s each, taking care not to touch the teeth, and the swabs were then placed in the same 50 mL Falcon tube. The 50 mL Falcon tube with the four swabs was then vortexed for 30 s and incubated at 4 °C for 24 h. After initial incubation, all samples were stored at − 80 °C until DNA extraction.

### Nicotine and cotinine analysis

Nicotine and cotinine analyses were performed to validate tobacco exposures reported on the tobacco use questionnaire. For each participant, 1 mL of saliva was spiked with internal standards (Nicotine-d 4 and Cotinine-d 3) and cleaned using solid-phase extraction (SPE) methods. All samples were analyzed for nicotine and cotinine using positive electrospray ionization (ESI +) isotope dilution liquid chromatography–tandem mass spectrometry (ID–LC–MS/MS) methods on an Applied Biosystems ABI3000 coupled with Shimadzu HPLC systems. Quantitation was performed based on selective reaction monitoring (SRM) transitions for the analytes (163 → 130 and 177 → 80 for nicotine and cotinine, respectively) as well as their internal standards (167 → 121 and 180 → 80 for Nicotine-d 4 and Cotinine-d 3, respectively)^77^. All results were adjusted for recovery rates and laboratory blanks. Samples with concentrations below the limit of detection (LOD) were assigned a value equivalent to ½ the limit of detection (LOD) following an established practice^78^.

### DNA extraction and 16S rRNA gene sequencing

500 µL of ice-cold 1X Phosphate-buffered saline (PBS; Thermo Fisher, MA) was added to 500 µL of each saliva sample and briefly vortexed. These saliva sample tubes, along with the buccal swab sample tubes, containing 1000 µL of the buccal swab solution, were centrifuged at 10,000 rpm for 30 min. The supernatant was discarded and 1 mL of ice-cold 1X PBS was then added to the cells left in the tubes. Negative control samples contained 1X PBS and no human samples. Total genomic DNA was then extracted from both saliva and buccal swab samples using previously published protocols (using both enzymatic and mechanical lysis approaches) followed by DNA purification with the Qiagen DSP DNA extraction kit (Qiagen, MD) per the manufacturer’s protocol^79^. DNA quality checks were then performed using Nanodrop spectrophotometric measurements and gel electrophoresis. PCR amplification of the V3-V4 hypervariable region of the 16S rRNA gene was then performed using the 319 F (ACTCCTACGGGAGGCAGCAG) and 806R (GGACTACHVGGGTWTCTAAT) universal primers and resulting amplicons were sequenced on an Illumina MiSeq 300 bp PE platform (Illumina, San Diego, CA) using a duel-indexing technique developed and validated at the Institute for Genomic Sciences^80,81^.

### Sequence quality filtering

After sequencing, 16S rRNA paired-end read pairs were assembled using PANDAseq^82^, demultiplexed and trimmed of artificial barcodes and primers. Next, the reads were trimmed for chimera using UCHIME^83^ and then incorporated into QIIME v1.9^84^. Quality reads were then clustered de-novo using VSEARCH^85^ into operational taxonomic units (OTUs) and taxonomies were assigned using the Greengenes database, using a 0.97 confidence threshold. The resulting OTU table, reference sequences, and phylogenetic tree files were then imported into R Statistical computing software (v. 0.99.473) using the Phyloseq R package (1.22.3)^86^.

### Statistical analysis

Alpha diversity was estimated using the *phyloseq* package (v. 1.19.1) (with Shannon indices and Observed number of species metrics) after rarefaction at a minimum depth of 2301 sequences for all samples. Statistical analyses were carried out using analysis of variance (ANOVA) and Tukey’s honestly significant difference (HSD) post hoc test at a 95% confidence level to measure variation among the samples within each group: *p-*values less than 0.05 were considered statistically significant. Cumulative sum scaling (CSS) was carried out to normalize reads using the *MetagenomeSeq* (v. 1.16.0) package^87^. Beta diversity was estimated using *vegan* v. 2.4.5 and *phyloseq* packages. Beta diversity was calculated using principal coordinate analysis (PCoA) and Bray–Curtis dissimilarity. Distances were tested for significance using Adonis (permutational multivariate analysis of variance) tests on 999 permutations between groups of samples. Relative abundances of bacterial taxa were compared across the sample types and user groups using the Kruskal–Wallis test. Statistical differences (*p* < 0.05) among bacterial OTUs relative abundances between samples were calculated using the DESeq2 package (at α = 0.001) on OTUs present at greater than 0.1% relative abundance^88^. Data were visualized with RStudio (v. 1.1.383) and the R package *ggplot2* (v. 2.2.1).

### Supplementary Information

Supplementary Information.

## Data Availability

Data generated from the samples included in this study are deposited in the NCBI BioProject database under accession number PRJNA690163.
