# Supplementary material for: Oral microbiome dysbiosis among cigarette smokers and smokeless tobacco users compared to non-users
Source: Sci Rep. 2024 May 6;14:10394. doi: 10.1038/s41598-024-60730-2 (PMC11074290; doi:10.1038/s41598-024-60730-2)
Supplement: Supplementary file 1 — Supplementary Information. [file 41598_2024_60730_MOESM1_ESM.docx]

**Supplementary Material**

# **Oral microbiome dysbiosis among cigarette smokers and smokeless tobacco users compared to non-users**

Suhana Chattopadhyay^1^, Leena Malayil^1^, Jessica Chopyk^1^, Eoghan Smyth^1^, Prachi Kulkarni^1^, Greg Raspanti^1^, Stephen B. Thomas^2^, Amir Sapkota^3^, Emmanuel F. Mongodin^4#^, Amy R. Sapkota^1*^

* Corresponding author: Amy R. Sapkota, [ars@umd.edu](mailto:ars@umd.edu)

**Sample size calculation**

The publicly available data from the Human Microbiome Project (HMP) Consortium authored published article (PMCID: PMC3564958), serves as a resource for power-analyses by allowing for empirical quantification of the variability of the healthy oral microbiome. They obtained taxonomic profiles of 233 HMP oral 16S datasets (131 buccal,102 saliva) and examined the variability of genera across the sampled population. They identified 30 genus-level groups (14 in buccal, 16 in saliva) that represented at least 1% of 16S sequences in the average oral microbiome. They measured the associated variance in relative abundance for each of these 30 genera and used the empirical results to perform a comprehensive power-analysis. For each genus, they estimated the power for a range of proportional shifts in average relative abundance (e.g. 0.50, 1.00, 2.00) and sample number. As expected, increased statistical power was found with higher abundance genera, so here we focus on power results for organisms reflecting an average of 1-3% of 16S sequences in the healthy oral microbiome. Among the 16 genera found at this low level in the oral HMP data, assuming equal variance in two normally-distributed treatment populations, and requiring a significance level of α=0.05, we expect that with at least 20 samples per treatment group, we will be able to detect a relative increase of a factor of 2 (e.g. 1% mean abundance increased to 3%), with statistical power of 97%. Given this estimate, we recruited 20 + (20% (20)) (to account for loss-to-follow- up) = 24 study participants per group.

**1 Supplementary Figures**

**Supplementary Figure 1.** Box plots of nicotine and cotinine levels detected in saliva samples from all participants, across all four-time points (T1 – T4). Cigarette user (CG, orange), smokeless tobacco user (ST, green), and non-user (NU)

**Supplementary Figure 2.** Beta diversity visualized through PCoA plots of Bray- Curtis computed distances among time points. Ellipses are drawn at 95% confidence intervals. User groups are displayed by circles and time points are displayed with dashed lines.

**Supplementary Figure 3.** Average relative abundance (± SE) of the top six bacterial genera present in all samples across four time points.

**Supplementary Figure 4.** Beta diversity visualized through PCoA plots of Bray- Curtis computed distances among races. Ellipses are drawn at 95% confidence intervals.

**Supplementary Figure 5.** Average relative abundance (± SE) of the top 15 bacterial genera present in all samples across the five races.

**Supplementary Figure 6.** Beta diversity was visualized through PCoA plots of Bray- Curtis computed distances among genders. Ellipses are drawn at 95% confidence intervals.

**Supplementary Figure 7.** Average relative abundance (± SE) of the top 15 bacterial genera present in all samples across two genders.

**Supplementary Figure 8.** Average relative abundance (± SE) of the top bacterial phyla present in all samples. Relative abundance was plotted for user groups (cigarette user (CG), orange; smokeless tobacco user (ST), green; non-user (NU), grey) from all sample types (buccal swab and saliva) across all time points.

**Supplementary Figure 9.** Box plots of relative abundance of the top 14 bacterial genera by sample type (buccal swab (red), saliva (blue) across each user group: cigarette users (CG), smokeless tobacco users (ST), and non-users (NU).
